# The additive effects of *GS3* and *qGL3* on rice grain length regulation revealed by genetic and transcriptome comparisons

**DOI:** 10.1186/s12870-015-0515-4

**Published:** 2015-06-24

**Authors:** Xiuying Gao, Xiaojun Zhang, Hongxia Lan, Ji Huang, Jianfei Wang, Hongsheng Zhang

**Affiliations:** State Key Laboratory of Crop Genetics and Germplasm Enhancement/Jiangsu Collaborative Innovation Center for Modern Crop Production, Nanjing Agricultural University, Nanjing, 210095 China; College of Agronomy and Plant Protection, Qingdao Agricultural University, Qingdao, 266109 China

**Keywords:** Additive effect, Grain length, *GS3*, *qGL3*, Rice, Transcriptome, Brassinosteroid

## Abstract

**Background:**

Grain length, as a critical trait for rice grain size and shape, has a great effect on grain yield and appearance quality. Although several grain size/shape genes have been cloned, the genetic interaction among these genes and the molecular mechanisms of grain size/shape architecture have not yet to be explored.

**Results:**

To investigate the genetic interaction between two major grain length loci of rice, *GS3* and *qGL3*, we developed two near-isogenic lines (NILs), NIL-*GS3* (*GS3*/*qGL3*) and NIL-*qgl*3 (*gs3*/*qgl3*), in the genetic background of 93–11 (*gs3*/*qGL3*) by conventional backcrossing and marker-assisted selection (MAS). Another NIL-*GS3*/*qgl3* (*GS3/qgl3*) was developed by crossing NIL-*GS3* with NIL-*qgl3* and using MAS. By comparing the grain lengths of 93–11, NIL-*GS3*, NIL-*qgl3* and NIL-*GS3*/*qgl3*, we investigated the effects of *GS3*, *qGL3* and *GS3* × *qGL3* interaction on grain length based on two-way ANOVA. We found that *GS3* and *qGL3* had additive effects on rice grain length regulation. Comparative analysis of primary panicle transcriptomes in the four NILs revealed that the genes affected by *GS3* and *qGL3* partially overlapped, and both loci might be involved in brassinosteroid signaling.

**Conclusion:**

Our data provide new information to better understand the rice grain length regulation mechanism and help rice breeders improve rice yield and appearance quality by molecular design breeding.

**Electronic supplementary material:**

The online version of this article (doi:10.1186/s12870-015-0515-4) contains supplementary material, which is available to authorized users.

## Background

When breeding cereal crops, the choice of a larger grain can increase the yield of crop varieties when other yield-related traits remain relatively stable. Among the three key components of rice yield (grain weight, panicles per plant and grain number per panicle), grain weight has high heritability [1]. Rice grains display a comparatively geometric shape, which can be broken down into grain length (GL), grain width (GW) and grain thickness (GT). These size/shape traits combined with grain density can explain the rice grain weight trait effectively.

Through linkage and association mapping, many quantitative trait loci (QTLs) for grain size/shape have been identified in different mutants or natural populations [2]. Only a small portion of these loci have been cloned, including *GS3* [3–5], *GL3.1*/*qGL3* [6, 7] and *TGW6* [8] for grain length, and *GW2* [9], *GW5*/*qSW5* [10, 11], *GS5* [12] and *GW8* [13] for grain width. Some grain size/shape QTLs, such as *gw8.1* [14], *GW6* [15], *qGL7* [16], *qGL7-2* [17], *GS7* [18] and *qSS7* [19], were also mapped to a narrow chromosome region. Additionally, several small (or short) seed phenotype causal genes were identified by map-based cloning, including *D1* [20–22], *BU1* [23], *SRS1* [24], *SRS3* [25], *SRS5* [26], and *SG1* [27].

There are few reports about the genetic interaction of these characterized genes [2]. Yan et al. (2011) found genetic interactions between *GS3* and *qSW5*. The effect of *qSW5* on seed length was masked by *GS3* alleles, and the effect of *GS3* on seed width was masked by *qSW5* alleles. No significant QTL interaction was observed between the two major grain width genes, *GW2* and *qSW5*/*GW5*, suggesting that they might work to regulate grain width in independent pathways [28]. *GS7* was effective in the presence of the *GS3* non-functional A-allele and ineffective when combined with the functional *GS3* C-allele [18]. However, how these genes work together or interact with others has not been deeply explored. The genetic interaction between two major grain length QTLs, *GS3* and *qGL3*, also remains unclear. At least four different alleles for *GS3* were identified by Mao et al. (2010): *GS3-1* (Zhenshan 97), *GS3-2* (Nipponbare), *GS3-3*/*gs3* (Minghui 63) and *GS3-4* (Chuan 7). *GS3-1* and *GS3-2* are functional short grain alleles, and *GS3-4* is a stronger functional extra-short grain forming allele. *GS3-3* has a premature termination, resulting in a non-functional long grain allele. At the cellular level, *GS3* controls grain size largely by modulating the longitudinal cell number in grain glumes. Its organ size regulation domain in the N-terminus is necessary and sufficient for it to function as a negative regulator and act as a dominant allele [3]. One of its homologs in the rice genome, *DENSE AND ERECT PANICLE1*, also functions as a negative regulator of rice grain length [29, 30]. Recently, its homolog in *Arabidopsis*, *AGG3*, was shown to be an atypical heterotrimeric GTP-binding protein (G-protein) γ-subunit that positively regulated organ size [31, 32]. Another major grain-length locus, *GL3.1*/*qGL3*, was map-based cloned and characterized by two independent groups [6, 7]. *GL3.1*/*qGL3* encodes a putative protein phosphatase (OsPPKL1) containing two Kelch domains. Transgenic studies showed that the Kelch domains functioned as a negative regulator and were essential for the biological function of OsPPKL1. At the cellular level, *qGL3* functions by negatively modulating the longitudinal cell number in grain glumes.

In this study, we focused on the genetic interaction between two major grain length QTLs, *GS3* and *qGL3*. The functional and non-functional alleles of *GS3* and *qGL3* were individually or simultaneously placed in the genetic background of 93–11 (an *indica* rice cultivar) to evaluate their genetic interaction. To understand these interactions at the molecular level, we analyzed the transcriptomes of young panicles (3–6 cm, glume development stage) of the NILs combining different alleles of *GS3* and *qGL3* through microarray assays. Our work could be helpful to better understand the genetic and molecular mechanisms of grain length regulation and molecular design rice breeding.

## Results

### The additive effects of *GS3* and *qGL3* on grain length

**Fig. 1:**
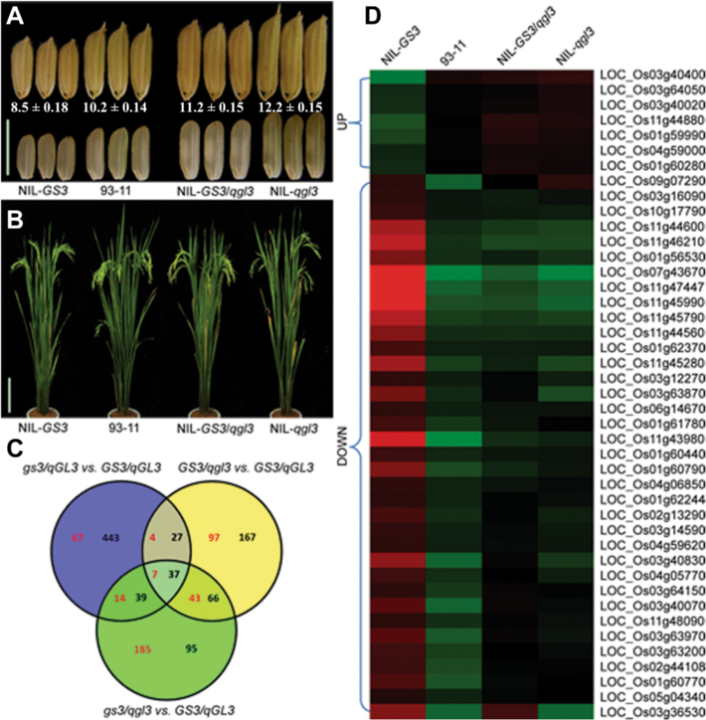
Grains and plants of the NILs and comparison of their expression profiles. **a** Grains of the three NILs and their genetic background, 93–11. Scale bar, 10.0 mm. **b** Plants of three NILs and their genetic background, 93–11. Scale bar, 20.0 cm. **c** Venn diagram of the genes from different comparisons; red numbers indicate up-regulation, black indicates down-regulation. **d** Expression profiles of the genes commonly regulated by the comparisons *gs3/qGL3* vs. *GS3/qGL3*, *GS3/qgl3* vs. *GS3/qGL3* and *gs3/qgl3* vs. *GS3/qGL3*

Functional *GS3* and non-functional *qgl3* were introduced into the 93–11 genetic background (genotype *gs3*/*qGL3*) to generate NIL-*GS3* (genotype *GS3*/*qGL3*) and NIL-*qgl3* (genotype *gs3*/*qgl3*), respectively. By crossing NIL-*GS3* with NIL-*qgl3*, and marker-assisted selection (MAS), we created a third line, NIL-*GS3*/*qgl3* (genotype *GS3*/*qgl3*). The grain lengths of these three NILs and their recurrent parent 93–11 with different allele combinations of *GS3* and *qGL3* were analyzed (Fig. 1a). We applied a two-way analysis of variance (ANOVA) for grain length (four NILs) and genotype (*GS3* and *qGL3*), and observed significant additive effects on grain length for *GS3* × *qGL3* (*P* = 1.27 × 10^−8^), *qGL3* (*P* = 3.71 × 10^−13^), and *GS3* (*P* = 4.4 × 10^−15^) (Table 1). Considering NIL-*GS3* (*GS3*/*qGL3*) as the control background, the loss of *GS3* increased the grain length from 8.5 mm (*GS3*/*qGL3*) to 10.2 mm (*gs3*/*qGL3*), the loss of *qGL3* increased the grain length from 8.5 mm (*GS3*/*qGL3*) to 11.2 mm (*GS3*/*qgl3*), and the loss of both increased the grain length from 8.5 mm (*GS3*/*qGL3*) to 12.2 mm (*gs3*/*qgl3*). Loss of *qGL3* increased grain length more in the functional *GS3* background (~2.7 mm) than in the non-functional *gs3* background (~2.0 mm). Similarly, loss of *GS3* increased grain length more in the functional *qGL3* background (~1.7 mm) than in the non-functional *qgl3* background (~1.0 mm) (Table 2). According to these data, we concluded that *GS3* and *qGL3* had additive effects larger than genetic interaction on rice grain length regulation and that the effects of *qGL3* were stronger (Table 1).

**Table 1:** *qGL3* × *GS3* interactions resolved by two-way ANOVA for grain length

Trait	Variation	SS	MS	*df*	*F*	*P*-value
GL	*qGL3*	5.43	5.43	1	7407.29	3.71 × 10^−13^
	*GS3*	16.45	16.45	1	22452.51	4.4 × 10^−15^
	*qGL3* × *GS3*	0.39	0.39	1	537.57	1.27 × 10^−8^
	Error	0.0058	0.0007	8		

*qGL3* × *GS3*, *qGL3*-by-*GS3* interaction; SS, MS, *df*, *F*, and *P-*values are from two-way ANOVA

**Table 2:** **Grain length of the genetic background 93**–**11 and its three NILs**

NIL Name (Genotype)	Grain length (mm)	ΔGrain length (mm)
NIL-*GS3* (*GS3/qGL3*)	8.5 ± 0.18	--
93-11 (*gs3/qGL3*)	10.2 ± 0.14	~1.7
NIL-*GS3/qgl3* (*GS3/qgl3*)	11.2 ± 0.15	~2.7
NIL-*qgl3* (*gs3/qgl3*)	12.2 ± 0.15	~3.7

Data are presented as means ± standard error. Δ Grain length shows the difference in grain length compared with NIL-*GS3*

### The genetic interactions between *GS3* and *qGL3* on the expression levels of commonly regulated genes

Based on the microarray data, by comparing the differentially expressed genes in *gs3/qGL3* vs. *GS3/qGL3*, *GS3/qgl3* vs. *GS3/qGL3*, and *gs3/qgl3* vs. *GS3/qGL3*, we found that seven genes were commonly up-regulated by > 1.5-fold (Fig. 1C, D and Table 3) and 37 genes were down-regulated by < 0.67-fold (Fig. 1c, d). Using gene expression levels (in 93–11 and its three NILs) and genotype (*GS3* and *qGL3*) as the main factors, we applied a two-way ANOVA to the datasets from all four microarrays to identify the seven up-regulated genes significantly affected by *GS3* and *qGL3* (Table 3). There were significant *GS3* × *qGL3* interactions for the expression levels of the seven up-regulated genes with *P*-values < 0.05, except for Os03g40400 and Os04g59000 (Table 3). Based on two-way ANOVA analysis, we found a significant genetic interaction between *GS3* and *qGL3* according to the expression levels of the genes down-regulated by *GS3* and *qGL3* (Additional file 1: Table S7). Interestingly, the effects of *GS3* and *qGL3* on grain length was additive, on the expression levels of the commonly regulated genes it showed significant genetic interaction.

**Table 3:** ***qGL3*** **×** ***GS3*** **interactions resolved by two**-**way ANOVA for the expression level of commonly up**-**regulated genes**

MSU_Gene_Symbol	Variation	SS	MS	*df*	*F*	*P* value
LOC_Os11g44880	*qGL3*	2285	2285	1	104.14	7.29 × 10^−06^
	*GS3*	3436	3436	1	156.61	1.56 × 10^−06^
	*qGL3* × *GS3*	2001	2001	1	91.23	1.19 × 10^−05^
	Error	176	22	8		
LOC_Os03g40400	*qGL3*	452326	452326	1	325.25	9.17 × 10^−08^
	*GS3*	86328	86328	1	62.08	4.87 × 10^−05^
	*qGL3* × *GS3*	85	85	1	0.06	0.810638
	Error	11126	1391	8		
LOC_Os03g64050	*qGL3*	9804786	9804786	1	196.20	6.55 × 10^−07^
	*GS3*	5377938	5377938	1	107.61	6.45 × 10^−06^
	*qGL3* × *GS3*	871662	871662	1	17.44	0.003095
	Error	399791	49974	8		
LOC_Os01g59990	*qGL3*	45189064	45189064	1	750.84	3.39 × 10^−09^
	*GS3*	24588257	24588257	1	408.55	3.75 × 10^−08^
	*qGL3* × *GS3*	3695841	3695841	1	61.41	5.07 × 10^−05^
	Error	481476	60185	8		
LOC_Os04g59000	*qGL3*	4655	4655	1	7.46	0.025761
	*GS3*	23058	23058	1	36.98	0.000296
	*qGL3* × *GS3*	3165	3165	1	5.08	0.05433
	Error	4989	624	8		
LOC_Os01g60280	*qGL3*	5192	5192	1	170.59	1.12 × 10^−06^
	*GS3*	3204	3204	1	105.27	7 × 10^−06^
	*qGL3* × *GS3*	3152	3152	1	103.57	7.45 × 10^−06^
	Error	243	30	8		
LOC_Os03g40020	*qGL3*	57233	57233	1	719.34	4.02 × 10^−09^
	*GS3*	13718	13718	1	172.41	1.08 × 10^−06^
	*qGL3* × *GS3*	19992	19992	1	251.27	2.51 × 10^−07^
	Error	637	80	8		

*qGL3* × *GS3*, *qGL3*-by-*GS3* interaction; SS, MS, *df*, *F*, and *P-*values are from two-way ANOVA

Among the seven genes up-regulated (>1.5-fold) by both *gs3* and *qgl3* (Fig. 1d), we found some encoded receptor protein kinases that might operate in the same signaling pathways to increase grain length in rice and explain the additive effects of *gs3* and *qgl3*. Another commonly up-regulated gene, Os11g44880, was found to encode a kinesin-4, whose homolog, *SRS3* (kinesin-13), was reported to positively regulate rice grain length [25]. Among the genes commonly down-regulated by *gs3* and *qgl3* (Fig. 1d), we found that *gs3* and *qgl3* down-regulated a gene (Os07g43670) encoding a ribonuclease T2 family domain-containing protein by 46- and 34-fold, respectively.

### Profiling of gene up- and down-regulation and gene ontology analysis of DEGs in different genotypes

To reveal the genes affected by *gs3* and *qgl3*, we compared the transcriptomes of the primary panicles of 93–11 (*gs3*/*qGL3*) and its three NILs through microarray analysis. Compared with the NIL-*GS3* (*GS3*/*qGL3*) background, 92 genes were up-regulated by > 1.5-fold and 546 genes were down-regulated by < 0.67-fold in 93–11 (*gs3*/*qGL3*) (Fig. 1c). Comparing the transcriptomes of NIL-*GS3*/*qgl3* (*GS3*/*qgl3*) with those of NIL-*qgl3* (*gs3*/*qgl3*) and NIL-*GS3* (*GS3*/*qGL3*) as well as 93–11 (*gs3*/*qGL3*), we found that 11 genes were up-regulated (Additional file 1: Table S1) and 15 genes were down-regulated (Additional file 1: Table S2). Among the 11 commonly up-regulated genes, one gene (Os03g27530) showed 18.7-fold induction under the NIL-*GS3* (*GS3*/*qGL3*) background and 41.4-fold induction under the NIL-*qgl3* (*gs3*/*qgl3*) background. It encoded a putative serine carboxypeptidase of the peptidase S10 family (Additional file 1: Table S1). Furthermore, we analyzed the genes commonly up- and down-regulated by *qgl3* in both the NIL-*qgl3* (*gs3*/*qgl3*) and NIL-*GS3* (*GS3*/*qGL3*) backgrounds and found 33 up-regulated genes and 30 down-regulated genes (Additional file 1: Tables S3 and S4). By comparing the transcriptomes of the panicles of NIL-*qgl3* (*gs3*/*qgl3*) and NIL-*GS3* (*GS3*/*qGL3*), we found that 249 genes were up-regulated by > 1.5-fold and 237 were down-regulated by < 0.67-fold (Fig. 1c). Among these, we found a down-regulated gene, Os03g63970, encoding a GA20 oxidase involved in the GA pathway. We also discovered that some genes involved in BR signaling were differentially expressed, such as a glycogen synthase kinase (CGMC_GSK) family gene (Os05g04340) (Additional file 1: Table S6). The number of down-regulated genes was higher than the number of up-regulated genes for 93–11 and its three NILs.

To determine the identities of the differentially expressed genes (DEGs), we categorized them based on their known functions using gene ontology (GO) classifications. The DEGs between combination I (*GS3*/*qGL3* vs. *gs3*/*qGL3* and *GS3*/*qgl3* vs. *gs3*/*qgl3*), combination II (*GS3*/*qGL3* vs. *GS3*/*qgl3* and *gs3*/*qGL3* vs. *gs3*/*qgl3*) and combination III (*GS3*/*qGL3* vs. *gs3*/*qgl3*) were used to analyze the GO pathways. These genes were associated with diverse biological, molecular and cellular functions, as shown in Tables 4, 5 and 6. This functional grouping primarily serves to facilitate data visualization. The functional classifications of the DEGs regulated by *gs3* were mainly associated with metabolic processes, catalytic activity, and binding (Table 4). The gene Os03g27530, which is also called *OsSCP16*, was associated with the GO:0008152 and GO:0003824 classifications. Its homolog in *Arabidopsis thaliana* is BRS1, which might participate in the BR signaling pathway. Interestingly, we also found this gene in combination III. The DEGs regulated by *qgl3* were mainly associated with metabolic processes, cell parts, catalytic activity, and binding (Table 5). According to q-PCR verification, the gene Os02g56310 encoding a calcium-dependent protein kinase was tremendously up-regulated in NIL-*qgl3* (*gs3*/*qgl3*), NIL-*GS3*/*qgl3* (*GS3*/*qgl3*) and 93–11 compared with NIL-*GS3* (*GS3*/*qGL3*). Ca^2+^ sensor protein kinases are prevalent in most plant species including rice. *OsCPK31*, which also encodes a calcium-dependent protein kinase, played a significant role in the grain filling process and eventually reduced the crop duration in overexpression plants [33]. The DEGs regulated by both *gs3* and *qgl3* were associated with 51 GO terms, which included the GO terms of *gs3* and *qgl3* (Table 6). Of these GO terms in Table 6, many transcripts encoded proteins involved in cellular metabolic process such as NB-ARC domain containing protein, F-box domain containing protein, zinc ion binding proteins and calcium-dependent protein kinase isoform AK1. In addition to genes associated with cellular metabolic process, genes associated with Leucine-Rich-Repeat (LRR) family protein and the calcium/calmodulin depedent protein kinases were annotated with the GO term “signal transduction”. Os03g27530 and Os02g56310 were also among the DEGs regulated by *gs3* and *qgl3*. In addition, Os07g05880 encoding F-box domain and kelch repeat containing protein, overlapping expression of rice F-box protein encoding genes during floral transition as well as panicle and seed development [34]. These results indicated that *gs3* and *qgl3* might participate in the same or parallel signaling pathways to regulate grain length.

**Table 4:** **Significant functions of DEGs regulated by** ***gs3***

GO term	Description	Input	BG/Ref	***p***-**value**	**FDR**
GO: 0008152	Metabolic process	5	7746	0.018	0.018
GO: 0005488	Binding	8	8681	5.90E-05	0.00018
GO: 0003824	Catalytic activity	7	8329	0.00052	0.00078

GO terms, such as “biological process”, “molecular function” and “cellular component”, were identified using AGRIGO (http://bioinfo.cau.edu.cn/agriGO/index.php) with default significance levels (FDR *<* 0.05). Input, gene number in input list; BG/Ref, gene number in BG/Ref

**Table 5:** **Significant functions of DEGs regulated by** ***qgl3***

GO term	Description	Input	BG/Ref	p-value	FDR
GO:0008152	Metabolic process	12	7746	1.00E-05	0.00012
GO:0005488	Binding	13	8681	4.20E-06	3.50E-05
GO:0003824	Catalytic activity	10	8329	0.00083	0.0035
GO:0043169	Cation binding	5	2582	0.0037	0.0076
GO:0043167	Ion binding	5	2584	0.0037	0.0076

GO terms, such as “biological process”, “molecular function” and “cellular component”, were identified using AGRIGO (http://bioinfo.cau.edu.cn/agriGO/index.php) with default significance levels (FDR *<* 0.05). Input, gene number in input list; BG/Ref, gene number in BG/Ref

**Table 6:** **Significant functions of DEGs regulated by both** ***gs3*** **and** ***qgl3***

**GO term**	**Description**	**Input**	**BG/Ref**	***p***-**value**	**FDR**
GO:0050896	Response to stimulus	16	1462	1.90E-13	2.10E-11
GO:0006950	Response to stress	13	885	1.50E-12	8.50E-11
GO:0009987	Cellular process	28	8160	1.50E-11	5.60E-10
GO:0008152	Metabolic process	24	7746	1.20E-08	3.40E-07
GO:0044238	Primary metabolic process	21	6775	1.90E-07	4.30E-06
GO:0065007	Biological regulation	12	2280	1.00E-06	1.90E-05
GO:0007165	Signal transduction	7	604	1.70E-06	2.80E-05
GO:0044237	Cellular metabolic process	19	6475	2.40E-06	3.40E-05
GO:0008219	Cell death	6	429	3.50E-06	4.00E-05
GO:0016265	Death	6	429	3.50E-06	4.00E-05
GO:0016310	Phosphorylation	8	1080	7.60E-06	6.80E-05
GO:0009719	Response to endogenous stimulus	5	277	7.30E-06	6.80E-05
GO:0019538	Protein metabolic process	12	2770	7.50E-06	6.80E-05
GO:0006796	Phosphate metabolic process	8	1206	1.70E-05	0.00013
GO:0006793	Phosphorus metabolic process	8	1206	1.70E-05	0.00013
GO:0006468	Protein amino acid phosphorylation	7	887	2.00E-05	0.00014
GO:0043687	Post-translational protein modification	8	1236	2.00E-05	0.00014
GO:0043170	Macromolecule metabolic process	16	5520	2.60E-05	0.00016
GO:0044267	Cellular protein metabolic process	10	2166	3.00E-05	0.00018
GO:0006464	Protein modification process	8	1359	3.90E-05	0.00023
GO:0043412	Macromolecule modification	8	1406	5.00E-05	0.00027
GO:0044260	Cellular macromolecule	14	4801	9.40E-05	0.00049
GO:0050789	Regulation of biological process	9	2112	0.00015	0.00073
GO:0016043	Cellular component organization	5	618	0.00031	0.0015
GO:0050794	Regulation of cellular process	8	1964	0.00048	0.0022
GO:0009058	Biosynthetic process	10	3129	0.0006	0.0026
GO:0001883	Purine nucleoside binding	15	1171	1.40E-13	4.80E-12
GO:0001882	Nucleoside binding	15	1171	1.40E-13	4.80E-12
GO:0030554	Adenyl nucleotide binding	15	1171	1.40E-13	4.80E-12
GO:0017076	Purine nucleotide binding	15	1317	7.30E-13	1.50E-11
GO:0005524	ATP binding	14	1071	8.20E-13	1.50E-11
GO:0032559	Adenyl ribonucleotide binding	14	1074	8.50E-13	1.50E-11
GO:0032555	Purine ribonucleotide binding	14	1218	4.50E-12	5.90E-11
GO:0032553	Ribonucleotide binding	14	1218	4.50E-12	5.90E-11
GO:0000166	Nucleotide binding	15	1686	2.30E-11	2.70E-10
GO:0005488	Binding	27	8681	5.00E-10	5.20E-09
GO:0003824	Catalytic activity	25	8329	8.60E-09	8.20E-08
GO:0004713	Protein tyrosine kinase activity	6	224	8.60E-08	7.50E-07
GO:0005515	Protein binding	11	1789	7.00E-07	5.60E-06
GO:0004871	Signal transducer activity	5	212	2.00E-06	1.40E-05
GO:0060089	Molecular transducer activity	5	212	2.00E-06	1.40E-05
GO:0016740	Transferase activity	12	3496	7.50E-05	0.00049
GO:0004672	Protein kinase activity	7	1102	7.90E-05	0.00049
GO:0016787	Hydrolase activity	10	2556	0.00012	0.00069
GO:0016773	Phosphotransferase activity	7	1238	0.00016	0.0009
GO:0004674	Serine/threonine kinase activity	6	949	0.00028	0.0015
GO:0016301	Kinase activity	7	1464	0.00044	0.0022
GO:0016491	Oxidoreductase activity	5	1141	0.0045	0.021
GO:0016772	Transferase activity, transferring	7	2197	0.0045	0.021
GO:0005886	Plasma membrane	9	494	1.00E-09	4.50E-08
GO:0016020	Membrane	12	4882	0.0016	0.036

GO terms, such as “biological process”, “molecular function” and “cellular component”, were identified using AGRIGO (http://bioinfo.cau.edu.cn/agriGO/index.php) with default significance levels (FDR *<* 0.05). Input, gene number in input list; BG/Ref, gene number in BG/Ref

### Metabolic pathways, cellular response and cell regulation analysis for DEGs

To identify genes related to metabolic reconfiguration in the different combinations, the MapMan tool was used to select and display the significantly regulated metabolic pathways. From our results, the up- and down-regulated genes were classified into 36 BINs.

**Fig. 2:**
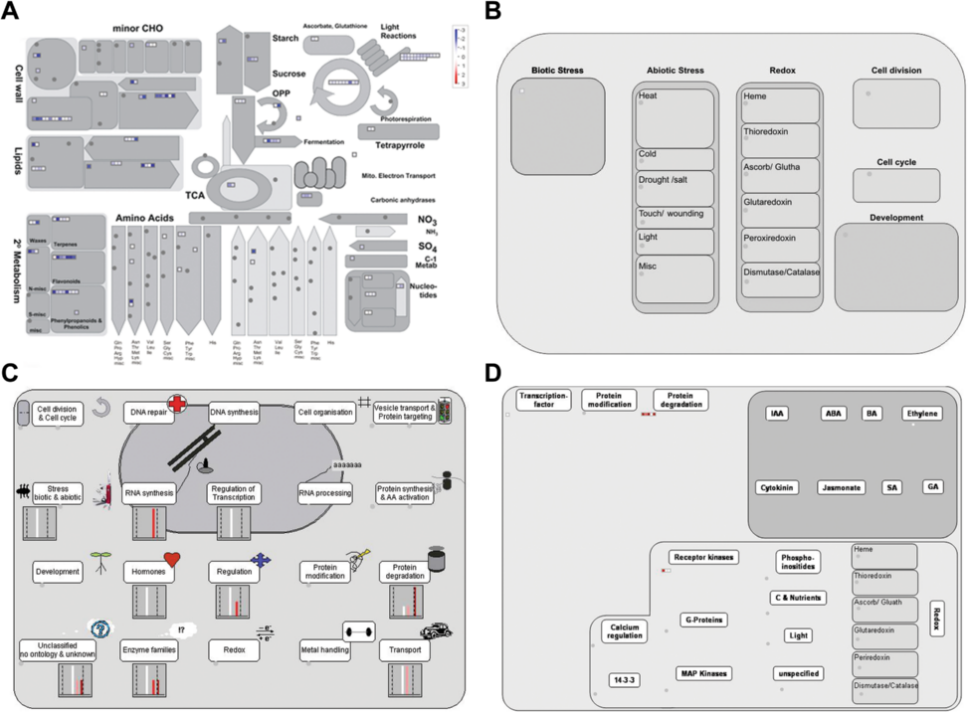
Overview of the differentially expressed genes between *GS3*/*qGL3* vs. *gs3*/*qGL3* and *GS3/qgl3* vs. *gs3/qgl3*. **a** Metabolism overview in MapMan. **b** Cellular response overview in MapMan. **c** Cell regulation overview in MapMan. **d** Regulation overview in MapMan. Red, up-regulation; white, no change; blue, down-regulation

**Fig. 3:**
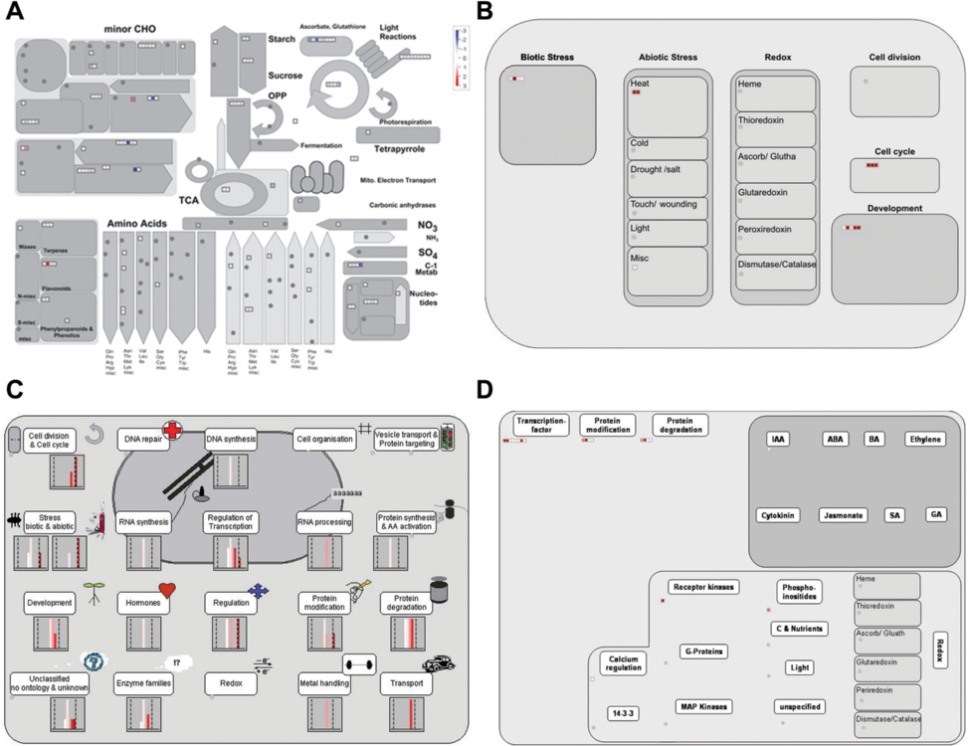
Overview of the differentially expressed genes between *GS3*/*qGL3* vs.*GS3*/*qgl3* and *gs3/qGL3* vs. *gs3/qgl3*. **a** Metabolism overview in MapMan. **b** Cellular response overview in MapMan. **c** Cell regulation overview in MapMan. **d** Regulation overview in MapMan. Red, up-regulation; white, no change; blue, down-regulation

**Fig. 4:**
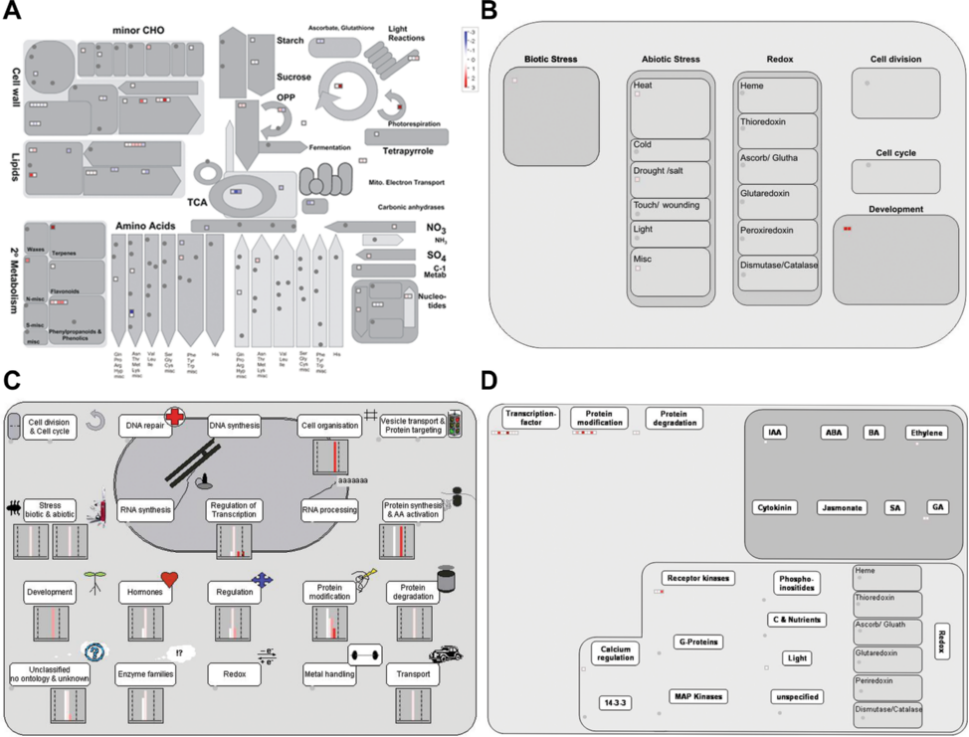
Overview of the differentially expressed genes between *GS3*/*qGL3* and *gs3*/*qgl3*. **a** Metabolism overview in MapMan. **b** Cellular response overview in MapMan. **c** Cell regulation overview in MapMan. **d** Regulation overview in MapMan. Red, up-regulation; white, no change; blue, down-regulation

By MapMan analysis of the DEGs regulated by *gs3*, we found that most of the genes associated with the cell wall, lipids, light reactions and secondary metabolism showed down-regulation (Fig. 2a). Some genes related to the cell wall were down-regulated by *gs3*, implying that down-regulation of these cell wall-related genes may negatively regulate cell wall formation. In our regulation overview, protein degradation and receptor kinases were the most frequent categories (Fig. 2d). In the hormone metabolism BIN, it was found that Os03g08500 was related with ethylene synsesis. Using the cell regulation and cell response overview function of MapMan, we found that genes related to protein degradation, biotic/abiotic stress, enzyme families, and transport were highly induced (Fig. 2c). In the protein degradation BIN, four up-regulated genes (Os03g28990, Os03g39230, Os03g27530 and Os03g37950) and one down-regulated gene (Os07g05880) were involved in it. Os03g27530 was in the protein degradation BIN and might participate in the BR signaling pathway. Os03g28990 encoding a von Willebrand factor type A (vWA) domain containing protein might regulate rice vegetative growth and development. However, in the cellular response overview we only found one gene (Os03g28190) related with biotic stress (Fig. 2b). DEGs associated with the cell wall, lipids, light reactions and secondary metabolism showed up-regulation, while some genes associated with the cell wall, lipids, and ascorbate and glutathione metabolism were down-regulated by *qgl3* (Fig. 3a). In the cellular response and cell regulation overview, genes related to hormones (auxin signal transduction), biotic/abiotic stress, RNA regulation of transcription, protein degradation, receptor kinase signaling, the cell cycle and protein modification were the most abundant (Fig. 3b, c). We further investigated three genes that were in the cell cycle BIN, Os02g55720, Os02g52360 and Os04g28420, all of which were up-regulated by *qgl3*. Os02g55720 encoded a kind of cyclin related to grain size regulation [6]. Os04g28420 encoded a kind of peptidyl-prolyl isomerase, which was up-regulated 17.97-fold by *qgl3* under the NIL-*gs3*/*qgl3* background (Additional file 1: Table S3). This indicated that *qGL3* might regulate grain length through regulation of the cell cycle. The regulation overview function of MapMan showed that DEGs associated with transcription factors, protein modification, and protein degradation were significantly regulated by *qgl3* (Fig. 3d). In the transcription factor BIN, it was found that some transcription factors, Os01g62130 encoding C2H2 zinc finger family protein, Os04g49450 encoding MYB related transcription and Os03g44540 encoding a CCAAT-box binding protein. The MapMan analysis indicated that some metabolic pathways were changed by allelic alterations at both loci, *GS3* and *qGL3* (Fig. 4a). We found that genes associated with photorespiration, light reactions, lipids, the cell wall and secondary metabolism were up-regulated, while genes related to lipids, the TCA cycle, and ascorbate and aldarate metabolisms were down-regulated (Fig. 4a). With cellular response overview, DEGs associated with biotic/abiotic stress and development were significantly regulated by both *gs3* and *qgl3* (Fig. 4b). DEGs in BINs such as transcription factors, protein modification, protein degradation, receptor kinases and hormones (ethylene, IAA and GA) were up-regulated by *gs3* and *qgl3* (Fig. 4c, d). In the GA synthesis overview, we found that a gene (Os03g63970) related with GA20 oxidase was down-regulated by both *gs3* and *qgl3*. It is possible that BR and GA interact closely to regulate cell elongation [35]. We found that some DEGs encoded regulators, including two transcription factors, a B3 DNA binding domain-containing protein (Os03g42370) and three MYB family transcription factor (Os06g14670, Os11g47460 and Os05g51160). These regulators might take part in the same signaling pathways to increase grain length in rice, which would explain the additive effects of *gs3* and *qgl3* (Additional file 1: Table S5). Overall, through MapMan analysis, we found that *gs3* and *qgl3* were involved in some common or parallel metabolic pathways to regulate grain length.

### Quantitative real-time PCR validation of DEGs

**Fig. 5:**
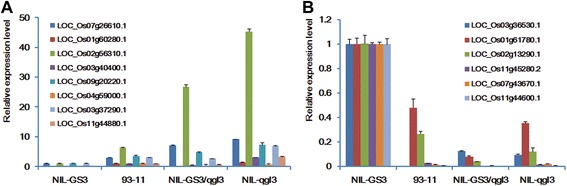
q-PCR validation of differentially expressed genes in the four rice lines. **a** Eight commonly up-regulated genes. **b** Six commonly down-regulated genes

To confirm the accuracy and reproducibility of the microarray results, eight genes commonly up-regulated and six genes commonly down-regulated by *gs3* and *qgl3* were selected for real-time PCR verification, including five BR signaling or grain length regulation associated genes, Os11g44880, Os07g43670, Os02g56310, Os01g43890 and Os01g60280. The q-PCR results for these genes were accordance with the microarray data (Fig. 5). The eight up-regulated genes and six down-regulated genes all showed up- and down-regulation in 93–11 (*gs3*/*qGL3*), NIL-*GS3*/*qgl3* (*GS3*/*qgl3*) and NIL-*qgl3* (*gs3*/*qgl3*) compared with the NIL-*GS3* (*GS3*/*qGL3*) background (Fig. 5). Strikingly, one gene, Os02g56310, encoding a calcium-dependent protein kinase, was obviously up-regulated in NIL-*qgl3* (*gs3*/*qgl3*), NIL-*GS3*/*qgl3* (*GS3*/*qgl3*) and 93–11 compared with NIL-*GS3* (*GS3*/*qGL3*) (Fig. 5A).

## Discussion

Grain size is a target in breeding and natural selection, and both *GS3* and *qGL3* significantly regulate grain size and organ size. In this study, we compared the grain lengths of four NILs, using NIL-*GS3* as a control group. The results indicated that *gs3* and *qgl3* had additive effects on rice grain length regulation. Moreover, *qGL3* had a stronger effect on rice grain length regulation than *GS3*. On grain length, the strength of the additive signal from *GS3* and *qGL3* was much larger than the genetic interaction signal. However, there were large genetic interactions between *GS3* and *qGL3* on the expression levels of commonly regulated genes rather than additive effects. This work represents the first analysis of the genetic interaction between *qGL3* and *GS3*. We used Gene Ontology [36] and MapMan [37] bioinformatics-based approaches for analyses aimed to interpret the biological significance of gene expression data. Through GO and MapMan analysis, we found that some genes regulated by *gs3* and *qgl3* are involved in BR signaling, the cell cycle, protein degradation, the GA/IAA family and protein modification, and might play important roles in the regulation of grain length. The *gs3* up-regulated gene, Os03g27530, was in the protein degradation BIN, and its homolog (*BRS1*) in *Arabidopsis* was reported to regulate BR signaling [38]. Os05g04340 in the protein modification BIN was down-regulated by both *gs3* and *qgl3*, and its homolog *BIN2* in *Arabidopsis* is a negative regulator of BR signaling [39]. Based on the functional annotations of the commonly regulated genes identified in this research, the regulation of grain length by *qGL3* and *GS3* might involve the BR signaling pathway.

BRs are a group of steroid phytohormones ubiquitously distributed throughout the plant kingdom [23]. They have essential roles in a wide range of plant growth and development processes, and can promote cell division or elongation and enhance tolerance to environmental stresses and resistance to pathogens [40]. The signal transduction pathway of BRs has been extensively studied [39]. The phosphorylation of BSK1 (BR-signaling kinase 1) by the BR receptor kinase BR-insensitive 1 (BRI1) promotes BSK1 binding to the BRI1 suppressor 1 (BSU1) phosphatase. BSU1, in turn, inactivates the GSK3-like kinase BR-insensitive 2 (BIN2) by dephosphorylating a conserved phospho-tyrosine residue (pTyr 200) [39, 41]. *qGL3* (*OsPPKL1*) encodes a protein phosphatase [7] and its two homologs in *Arabidopsis*, *BSU1* and *BSL1*, were reported to promote brassinosteroid signaling [39, 42]. They transmit a signal by dephosphorylating and deactivating the BIN2 kinase downstream of BR signaling [39]. Moreover, we found that genes involved in BR signaling, such as the *CGMC_GSK* family genes, encoding *Arabidopsis* BIN2 homologous proteins, were differentially expressed between NIL-*GS3* (*GS3*/*qGL3*) and NIL-*GS3*/*qgl3* (*gs3*/*qgl3*). Recently, we cloned the GSK family genes and obtained additional evidence for the interaction of OsPPKL1 and GSKs via yeast two-hybrid assays (unpublished data). These data indicated that *qGL3* might participate in BR signaling by dephosphorylating GSKs. However, *qGL3* is a negative regulator of rice grain length [7], suggesting that OsPPKL1-GSK interaction might play different roles in BR signaling in rice compared with BSU1- and BSL1-BIN2 interaction in *Arabidopsis*.

*GS3* is a major QTL for grain length and weight and a minor QTL for grain width and thickness [5]. GS3 was reported to be an atypical heterotrimeric G protein γ-subunit that positively regulates organ size [31, 32]. The heterotrimeric G protein α-subunit, known as D1/RGA1 in rice, is involved in an alternative BR-signaling pathway, independent of *OsBRI1*. Recently, Hu et al. (2013) reported that a U-Box E3 ubiquitin ligase worked as a linkage factor between the heterotrimeric Gα subunit and BR signaling to mediate rice growth, mainly by regulating cell proliferation and organizing cell files in aerial organs. In this study, we found that *gs3* up-regulated a putative serine carboxypeptidase of the peptidase S10 family. Its homolog in *Arabidopsis* (*BRS1*) was reported to positively regulate BR signaling [38]. We believe that this gene might have *GS5*-like properties. Overexpression of *BRS1* suppressed the cell surface receptor for BRs in *bri1* extracellular domain mutants [38]. One of its homologs in rice was cloned as the grain-size gene *GS5*, which increased grain width when its expression increased [12]. These data reveal that some members of the serine carboxypeptidase family might act downstream of BR signaling as positive factors. Our research implies that *GS3* also takes some part in BR signaling, and both *GS3* and *qGL3* might share a common BR signaling associated pathway in the regulation of rice grain length. We suppose that *qGL3* might directly participate in brassinolide signaling by dephosphorylating GSKs, while *GS3* indirectly influences BRS1, which is parallel to the BRI-mediated BR signaling pathway.

Among the genes up-regulated by both loci, we found a gene encoding a kinesin-4, whose homolog *SRS3* was reported to positively regulate rice grain length in seed formation [25]. We identified a small and round seed mutant phenotype (*srs3*). The gene, which belongs to the kinesin 13 subfamily, was designated *SRS3* [25]. The shortened seed phenotype of the *srs3* mutant was probably the result of a reduction in cell length in the longitudinal direction [25]. The SRS3 protein might be a homolog of the AtKinesin 13A protein, which regulates trichome elongation in *Arabidopsis* [43]. Interestingly, among the genes commonly down-regulated by *gs3* and *qgl3*, we observed that a number of disease resistance related genes, encoding two NB-ARC domain containing proteins, a stripe rust resistance protein Yr10 and a peroxidase precursor, were down-regulated by both *qgl3* and *gs3*, suggesting that disease resistance responses may also be negatively correlated with grain development. In addition, we found a gene (Os07g43670) encoding a ribonuclease T2 family domain containing protein involved in the cytokinin signaling pathway. A major QTL, *Grain number 1a* (*Gn1a*), encodes a cytokinin oxidase/dehydrogenase (OsCKX2) that catalyzes the irreversible degradation of cytokinin. Mutation in *Gn1a/OsCKX2* [44], which encodes a zinc finger transcription factor that directly and positively regulates *Gn1a/OsCKX2* [2, 45], caused the accumulation of cytokinin and consequently increased grain number [2]. In many cases, increased grain number is closely associated with reduced grain size, likely owing to the availability of fixed carbon in the source and the efficiency of transport to the sink [7, 9, 29].

The currently available evidence suggests that the mechanisms underlying the additive effects of *GS3* and *qGL3* in regulating grain length might involve phytohormones (especially BRs) and key genes related to cell division or elongation. This research should help us to understand the mechanisms of the additive effects of *gs3* and *qgl3*, which would be useful for deciphering the genetic network involved in rice seed formation and for molecular breeding.

## Conclusions

With an elite *indica cultivar* 93–11 as recurrent parent NIL-*GS3* (*GS3/qGL3*) and NIL-*qgl3* (*gs3/qgl3*) were developed by conventional backcrossing and marker-assisted selection. Another line, NIL-*GS3/qgl3*, was developed by crossing NIL-*GS3* and NIL-*qgl3*. By comparing the grain length of 93–11 and its three NILs we concluded that *gs3* and *qgl3* had additive effects on rice grain length regulation and that the effects of *qGL3* were stronger. To reveal the genes affected by *gs3* and *qgl3*, we compared the transcriptomes of the primary panicles of 93–11 and the three NILs through microarray analysis. The transcriptome analysis revealed that the genes affected by *GS3* and *qGL3* partially overlapped, and both loci might be involved in BR signaling.

## Methods

### Plant materials and development of the NILs

The high-quality, previously sequenced [46] elite *indica* rice cultivar 93–11 with non-functional *gs3* and functional *qGL3* was used as the genetic background for introducing the functional *GS3* and non-functional *qgl3* alleles. The *japonica* rice cultivar Koshihikari was used as the donor parent for functional *GS3*. The *GS3* allele in Koshihikari was cloned and sequenced, and was found to be the same as *GS3-2* (Nipponbare) [3, 18]. The rice accession N411 with extra-large grains was used as the donor parent for non-functional *qgl3* [7].

As the functional *GS3* is a dominant allele forming short grains, plants with 93-11-like performance with short grains were selected from BC_n_F_1_ populations of 93–11 × Koshihikari and continuously backcrossed with 93–11. To develop NIL-*GS3* (genotype *GS3/qGL3*), we selected plants, from the BC_4_F_1_ population, with a short Koshihikari segment (from RM15144 to RM411) and the *GS3* allele for self-pollination. A total of 126 simple sequence repeat markers were employed for background detection. NIL-*qgl3* (genotype *gs3*/*qgl3*), which carries a ~113-kb segment, including the N411 *qgl3* allele in the 93–11 background, was described in previous studies [7]. NIL-*GS3*/*qlg3* (genotype *GS3/qgl3*) was developed by crossing NIL-*GS3* and NIL-*qgl3*. In the NIL-*GS3* × NIL-*qgl3* F_2_ population, plants heterozygous at the *GS3* locus and homozygous at the *qgl3* locus were selected to self-pollinate naturally and the homozygous NIL-*GS3*/*qlg3* was selected from the F_3_ family by MAS.

### Plant growth and evaluation of agronomic traits

To evaluate the differences in grain length between the recurrent parent 93–11 and its three NILs, all materials including 93–11, NIL-*qgl3*, NIL-*GS3* and NIL-*GS3*/*qgl3* were grown in the Jiangpu Experiment Station of Nanjing Agricultural University. The four materials were grown in a 13.4-m^2^ acreage (the actual used area: 1.5 m × 8.0 m). All experimental materials were transplanted in the fields with 15 cm spacing between plants within rows and 25 cm spacing between rows. The 13.4-m^2^ block was divided into four plots (the area of one plot: 1.5 m × 2 m), with 80 plants of each material in one plot and there were three blocks. 10 plants selected randomly from 80 plants of each material were measured. The mean value of the 10 plants was used for analysis. T-test was carried out to evaluate the statistical differences in their grain length between NIL-*GS3* and other three materials. Grain length was measured as described in a previous study [7].

### Microarray analysis

As reported in previous studies, *GS3* and *qGL3* are expressed strongly in young panicles [4, 7]. Thus, we used primary panicles of 3–6 cm length from the three NILs and 93–11 for RNA preparation and hybridization with the Rice Genome OneArray Microarray (Phalanx Biotech Group, Hai Shang). Each NIL and 93–11 was sampled three times from different tillers. The Rice OneArray probe was set with a combination of the Rice Genome Annotation Project (RGAP) version 6.1 and Beijing Genomics Institute (BGI) version 2008 databases. Long oligonucleotide probes (~60-mers) were engineered using specific lengths to match their melting temperatures for superior hybridization performance. Each microarray contained 824 performance monitoring control probes for hybridization, sample quality, and labeling reactions. RNA isolation, purification and microarray hybridization were conducted by the Phalanx Biotech Group. Longer grains were regarded as being more active during the growth of the grain or glume. We conducted a comparison of the transcriptomes by comparing the longer grain genotype with the shorter grain genotype. The microarray data were normalized using the GC-RMA algorithm followed by *Log*_*2*_ transformation. We used ordinary Student’s t –test (*P* value < 0.05) to identify significantly differentially expressed genes. Probe sets showing more than 1.5-fold change (four NILs) in expression were considered as DEGs. To identify DEGs regulated by *gs3* or *qgl3*, we used the ratio (1.5 folds for up-regulation and 0.67 folds for down-regulation) of the expression level between combinations *gs3*/*qGL3* vs. *GS3*/*qGL3, gs3*/*qgl3* vs. *GS3*/*qgl3, GS3*/*qgl3* vs. *GS3*/*qGL3, and gs3*/*qgl3* vs. *gs3*/*qGL3.* To identify commonly expressed genes in the four materials, we used the ratio (1.5 folds for up-regulation and 0.67 folds for down-regulation) of the expression level between combination *gs3/qGL3* vs. *GS3/qGL3*, *GS3/qgl3* vs. *GS3/qGL3*, and *gs3/qgl3* vs. *GS3/qGL3*. A two-way analysis of variance (ANOVA) with expression levels and genotype (*GS3*/*gs3* and *qGL3*/*qgl3*) as main factors was applied to the datasets from all four microarrays to identify genes significantly affected by *GS3*, *qGL3*, or *GS3* × *qGL3* interaction. The Benjamini–Hochberg false discovery rate (FDR) for multiple test correction was used for the analysis [47]. Furthermore, the statistical criterion of at least a 1.5-fold change at a *P*-value ≤ 0.05 was used for gene selection.

### Pathway analysis

Functional enrichment analysis of DEGs using the GO domains “molecular function”, “biological process” and “cellular component” was performed using the AGRIGO website with a significance level of FDR *<* 0.05 [36]. The MapMan tool [37] was employed to analyze the metabolic and signaling changes in the microarray data based on the expression value of each DEG. A metabolic pathway overview was produced by loading the DEGs with their *Log2* expression values into the locally-installed MapMan program and shown using color intensity.

### Real-time quantitative PCR

Based on the transcriptome comparison between the three NILs and 93–11, several DEGs were selected for further confirmation by real-time quantitative PCR. Primary panicles of 3–6 cm length were used for total RNA extraction with an RNA extraction kit (RNAiso Plus, TaKaRa Bio, Inc.). Reverse transcription was performed using 6 μg RNA and 4 μg reverse transcriptase mix (PrimeScript® RT Master Mix Perfect Real Time, TaKaRa Bio) in a volume of 40 μl, according to the manufacturer’s protocol. Real-time PCR was carried out in a total volume of 25 μl containing 2 μl of cDNA, 0.2 mM gene-specific primers, 12.5 μl SYBR® Premix Ex Taq TM II, and 0.5 μl of Rox Reference Dye II (TaKaRa Bio), using an ABI 7500 Fast Real-Time PCR System according to the manufacturer’s instructions. The rice 18S *rRNA* gene was used as an internal control. Relative quantification of the transcript levels was performed using the 2^−ΔΔCT^ method [48].

### Availability of supporting data

The microarray data for the four NILs has been submitted to the Gene Expression Omnibus (GEO; http://www.ncbi.nlm.nih.gov/geo/) under accession number GSE59619.

## Additional file

Additional file 1: Table S1. Genes up-regulated by *gs3* (>1.5-fold). **Table S2.** Genes down-regulated by *gs3* (<0.67-fold). **Table S3.** Genes up-regulated by *qgl3* (>1.5-fold). **Table S4.** Genes down-regulated by *qgl3* (<0.67-fold).** Table S5.** Genes up-regulated by both *qgl3* and *gs3* (<0.67-fold). **Table S6.** Genes down-regulated by both *qgl3* and *gs3* (<0.67-fold). Additional file 1: **Table S7.** *qGL3* × *GS3* interactions resolved by two-way ANOVA for the expression level of commonly regulated genes.

## Abbreviations

NIL: Near-isogenic lines
MAS: Marker-assisted selection
QTL: Quantitative trait locus
SSR: Simple sequence repeat
ANOVA: Analysis of variance
GL: Grain length
DEG: Differentially expressed gene
qRT-PCR: Quantitative reverse transcription polymerase chain reaction
GO: Gene ontology
FDR: False discovery rate

## References

[1] Xing YZ, Zhang QF (2010). Genetic and Molecular Bases of Rice Yield. Annu Rev Plant Biol.

[2] Zuo J, Li J. Molecular Genetic Dissection of Quantitative Trait Loci Regulating Rice Grain Size. Annu Rev Genet. 2014. DOI: 10.1146/annurev-genet-120213-092138. 10.1146/annurev-genet-120213-092138 25149369

[3] Mao HL, Sun SY, Yao JL, Wang CR, Yu SB, Xu CG (2010). Linking differential domain functions of the GS3 protein to natural variation of grain size in rice. Proc Natl Acad Sci U S A.

[4] Takano-Kai N, Jiang H, Kubo T, Sweeney M, Matsumoto T, Kanamori H (2009). Evolutionary History of *GS3*, a Gene Conferring Grain Length in Rice. Genetics.

[5] Fan CH, Xing YZ, Mao HL, Lu TT, Han B, Xu CG (2006). GS3, a major QTL for grain length and weight and minor QTL for grain width and thickness in rice, encodes a putative transmembrane protein. Theor Appl Genet.

[6] Qi P, Lin YS, Song XJ, Shen JB, Huang W, Shan JX (2012). The novel quantitative trait locus *GL3.1* controls rice grain size and yield by regulating Cyclin-T1;3. Cell Res.

[7] Zhang X, Wang J, Huang J, Lan H, Wang C, Yin C (2012). Rare allele of OsPPKL1 associated with grain length causes extra-large grain and a significant yield increase in rice. Proc Natl Acad Sci U S A.

[8] Ishimaru K, Hirotsu N, Madoka Y, Murakami N, Hara N, Onodera H (2013). Loss of function of the IAA-glucose hydrolase gene *TGW6* enhances rice grain weight and increases yield. Nat Genet.

[9] Song XJ, Huang W, Shi M, Zhu MZ, Lin HX (2007). A QTL for rice grain width and weight encodes a previously unknown RING-type E3 ubiquitin ligase. Nat Genet.

[10] Shomura A, Izawa T, Ebana K, Ebitani T, Kanegae H, Konishi S (2008). Deletion in a gene associated with grain size increased yields during rice domestication. Nat Genet.

[11] Weng JF, Gu SH, Wan XY, Gao H, Guo T, Su N (2008). Isolation and initial characterization of *GW5*, a major QTL associated with rice grain width and weight. Cell Res.

[12] Li Y, Fan C, Xing Y, Jiang Y, Luo L, Sun L (2011). Natural variation in *GS5* plays an important role in regulating grain size and yield in rice. Nat Genet.

[13] Wang SK, Wu K, Yuan QB, Liu XY, Liu ZB, Lin XY (2012). Control of grain size, shape and quality by OsSPL16 in rice. Nat Genet.

[14] Xie XB, Song MH, Jin FX, Ahn SN, Suh JP, Hwang HG (2006). Fine mapping of a grain weight quantitative trait locus on rice chromosome 8 using near-isogenic lines derived from a cross between *Oryza sativa* and *Oryza rufipogon*. Theor Appl Genet.

[15] Guo LB, Ma LL, Jiang H, Zeng DL, Hu J, Wu LW (2009). Genetic Analysis and Fine Mapping of Two Genes for Grain Shape and Weight in Rice. J Integr Plant Biol.

[16] Bai XF, Luo LJ, Yan WH, Kovi MR, Zhan W, Xing YZ: Genetic dissection of rice grain shape using a recombinant inbred line population derived from two contrasting parents and fine mapping a pleiotropic quantitative trait locus *qGL7. Bmc Genet* 2010;11(16):2187. 10.1186/1471-2156-11-16 PMC2846863 20184774

[17] Shao GN, Tang SQ, Luo J, Jiao GA, Wei XJ, Tang A (2010). Mapping of *qGL7-2*, a grain length QTL on chromosome 7 of rice. J Genet Genomics.

[18] Shao GN, Wei XJ, Chen ML, Tang SQ, Luo J, Jiao GA (2012). Allelic variation for a candidate gene for *GS7*, responsible for grain shape in rice. Theor Appl Genet.

[19] Qiu X, Gong R, Tan Y, Yu S (2012). Mapping and characterization of the major quantitative trait locus *qSS7* associated with increased length and decreased width of rice seeds. Theor Appl Genet.

[20] Ashikari M, Wu JZ, Yano M, Sasaki T, Yoshimura A (1999). Rice gibberellin-insensitive dwarf mutant gene Dwarf 1 encodes the alpha-subunit of GTP-binding protein. Proc Natl Acad Sci U S A.

[21] Izawa Y, Takayanagi Y, Inaba N, Abe Y, Minami M, Fujisawa Y (2010). Function and Expression Pattern of the alpha Subunit of the Heterotrimeric G Protein in Rice. Plant Cell Physiol.

[22] Oki K, Inaba N, Kitano H, Takahashi S, Fujisawa Y, Kato H (2009). Study of novel d1 alleles, defective mutants of the alpha subunit of heterotrimeric G-protein in rice. Genes Genet Syst.

[23] Tanaka A, Nakagawa H, Tomita C, Shimatani Z, Ohtake M, Nomura T (2009). BRASSINOSTEROID UPREGULATED1, Encoding a Helix-Loop-Helix Protein, Is a Novel Gene Involved in Brassinosteroid Signaling and Controls Bending of the Lamina Joint in Rice. Plant Physiol.

[24] Abe Y, Mieda K, Ando T, Kono I, Yano M, Kitano H (2010). The SMALL AND ROUND SEED1 (*SRS1/DEP2*) gene is involved in the regulation of seed size in rice. Genes Genet Syst.

[25] Kitagawa K, Kurinami S, Oki K, Abe Y, Ando T, Kono I (2010). A Novel Kinesin 13 Protein Regulating Rice Seed Length. Plant Cell Physiol.

[26] Segami S, Kono I, Ando T, Yano M, Kitano H, Miura K, Iwasaki Y: Small and round seed 5 gene encodes alpha-tubulin regulating seed cell elongation in rice. *Rice* 2012;5(4):225. 10.1186/1939-8433-5-4 PMC3834490 24764504

[27] Nakagawa H, Tanaka A, Tanabata T, Ohtake M, Fujioka S, Nakamura H (2012). Short grain1 decreases organ elongation and brassinosteroid response in rice. Plant Physiol.

[28] Ying JZ, Gao JP, Shan JX, Zhu MZ, Shi M, Lin HX (2012). Dissecting the genetic basis of extremely large grain shape in rice cultivar ‘JZ1560’. J Genet Genomics.

[29] Huang X, Qian Q, Liu Z, Sun H, He S, Luo D (2009). Natural variation at the *DEP1* locus enhances grain yield in rice. Nat Genet.

[30] Zhou Y, Zhu J, Li Z, Yi C, Liu J, Zhang H (2009). Deletion in a quantitative trait gene *qPE9-1* associated with panicle erectness improves plant architecture during rice domestication. Genetics.

[31] Li S, Liu W, Zhang X, Liu Y, Li N, Li Y (2012). Roles of the *Arabidopsis* G protein gamma subunit AGG3 and its rice homologs GS3 and DEP1 in seed and organ size control. Plant Signal Behav.

[32] Li S, Liu Y, Zheng L, Chen L, Li N, Corke F (2012). The plant-specific G protein gamma subunit AGG3 influences organ size and shape in *Arabidopsis thaliana*. New Phytol.

[33] Manimaran P, Mangrauthia SK, Sundaram RM, Balachandran SM (2015). Constitutive expression and silencing of a novel seed specific calcium dependent protein kinase gene in rice reveals its role in grain filling. J Plant Physiology.

[34] Jain M, Nijhawan A, Arora R, Agarwal P, Ray S, Sharma P (2007). F-box proteins in rice. Genome-wide analysis, classification, temporal and spatial gene expression during panicle and seed development, and regulation by light and abiotic stress. Plant Physiol.

[35] Tong H, Xiao Y, Liu D, Gao S, Liu L, Yin Y (2014). Brassinosteroid regulates cell elongation by modulating gibberellin metabolism in rice. The Plant cell.

[36] Du Z, Zhou X, Ling Y, Zhang Z, Su Z (2010). agriGO: a GO analysis toolkit for the agricultural community. Nucleic acids research.

[37] Thimm O, Blasing O, Gibon Y, Nagel A, Meyer S, Kruger P (2004). MAPMAN: a user-driven tool to display genomics data sets onto diagrams of metabolic pathways and other biological processes. Plant J.

[38] Li J, Lease KA, Tax FE, Walker JC (2001). BRS1, a serine carboxypeptidase, regulates BRI1 signaling in *Arabidopsis thaliana*. Proc Natl Acad Sci U S A.

[39] Kim TW, Guan SH, Sun Y, Deng ZP, Tang WQ, Shang JX (2009). Brassinosteroid signal transduction from cell-surface receptor kinases to nuclear transcription factors. Nat Cell Biol.

[40] Zhang C, Bai MY, Chong K (2014). Brassinosteroid-mediated regulation of agronomic traits in rice. Plant Cell Rep.

[41] Kim TW, Wang ZY (2010). Brassinosteroid Signal Transduction from Receptor Kinases to Transcription Factors. Annual RevPlant Biol.

[42] Mora-Garcia S, Vert G, Yin Y, Cano-Delgado A, Cheong H, Chory J (2004). Nuclear protein phosphatases with Kelch-repeat domains modulate the response to brassinosteroids in *Arabidopsis*. Gene develop.

[43] Lu L, Lee YRJ, Pan RQ, Maloof JN, Liu B (2005). An internal motor kinesin is associated with the golgi apparatus and plays a role in trichome morphogenesis in *Arabidopsis*. Mol Biol Cell.

[44] Ashikari M, Sakakibara H, Lin SY, Yamamoto T, Takashi T, Nishimura A (2005). Cytokinin oxidase regulates rice grain production. Science.

[45] Li SY, Zhao BR, Yuan DY, Duan MJ, Qian Q, Tang L (2013). Rice zinc finger protein DST enhances grain production through controlling *Gn1a/OsCKX2* expression. Proc Natl Acad Sci U S A.

[46] Yu J, Hu S, Wang J, Wong GK, Li S, Liu B (2002). A draft sequence of the rice genome (*Oryza sativa* L. ssp. *indica*). Science.

[47] Benjamini Y, Hochberg Y (1995). Controlling the False Discovery Rate - a Practical and Powerful Approach to Multiple Testing. J Roy Stat Soc B Met.

[48] Livak KJ, Schmittgen TD (2001). Analysis of relative gene expression data using real-time quantitative PCR and the 2(T)(−Delta Delta C) method. Methods.

