# Supplementary material for: The additive effects of GS3 and qGL3 on rice grain length regulation revealed by genetic and transcriptome comparisons
Source: BMC Plant Biol. 2015 Jun 24;15:156. doi: 10.1186/s12870-015-0515-4 (PMC4479070; doi:10.1186/s12870-015-0515-4)
Supplement: Additional file 1: Table S1. — Genes up-regulated by gs3 (>1.5-fold). Table S2. Genes down-regulated by gs3 (<0.67-fold). Table S3. Genes up-regulated by qgl3 (>1.5-fold). Table S4. Genes down-regulated by qgl3 (<0.67-fold). Table S5. Genes up-regulated by both qgl3 and gs3 (<0.67-fold). Table S6. Genes down-regulated by both qgl3 and gs3 (<0.67-fold). Additional file 1: Table S7. qGL3 × GS3 interactions resolved by two-way ANOVA for the expression level of commonly regulated genes. [file 12870_2015_515_MOESM1_ESM.pdf]

Supplemental Table S1 Genes up-regulated by *gs3* (> 1.5-fold).

MSU_Gene_Symbol*	*gs3*/*GS3* (*qGL3*;fold)	*gs3*/*GS3* (*qgl3*;fold)	Description
LOC_Os03g28990	21.04	35.77	zinc finger family protein, putative, expressed
LOC_Os03g27530	18.82	41.43	OsSCP16 - Putative Serine Carboxypeptidase homologue
LOC_Os03g32050	4.93	17.80	peroxidase precursor, putative, expressed
LOC_Os03g37950	3.69	4.10	proteasome subunit, putative, expressed
LOC_Os03g37290	2.33	2.46	cytochrome P450, putative, expressed
LOC_Os03g28270	5.04	2.79	Leucine Rich Repeat family protein, expressed
LOC_Os03g28400	2.04	2.12	transport protein particle component, Bet3, domain containing protein, expressed
LOC_Os03g32040	3.06	3.13	phenazine biosynthesis protein, putative, expressed
LOC_Os03g39230	2.29	1.97	OTU-like cysteine protease family protein, putative, expressed
LOC_Os03g38960	2.01	2.42	DNA-directed RNA polymerase subunit, putative, expressed
LOC_Os03g37090	1.54	1.70	expressed protein

*These genes located in Chr. 3 but not the segments harbors the different segments from Koshihikari (from RM15144 to RM411 positions from Chr3. 17,679,472 to 21,231,694) or N411 (from XJ19 to RM15575 positions from Chr3. 24953205 to 25099651) checked with MSU Rice Genome Annotation (Osa1) Release 7.

Supplemental Table S2 Genes down-regulated by *gs3* (＜0.67-fold)

MSU_Gene_Symbol*	*gs3*/*GS3* (*qGL3*;fold)	*gs3*/*GS3* (*qgl3*;fold)	Discription
LOC_Os03g32470	0.01	0.01	leucoanthocyanidin dioxygenase, putative, expressed
LOC_Os07g05640	0.11	0.09	transporter family protein, putative, expressed
LOC_Os03g37580	0.13	0.12	modifier of rudimentary protein, expressed
LOC_Os07g03990	0.16	0.09	leucine-rich repeat family protein, putative, expressed
LOC_Os06g01890	0.22	0.19	MADS-box transcription factor, putative, expressed
LOC_Os04g04060	0.32	0.43	dynamin family protein, putative, expressed
LOC_Os07g05880	0.32	0.37	OsFBK18 - F-box domain and kelch repeat containing protein, expressed
LOC_Os03g31070	0.35	0.30	protein kinase, putative, expressed
LOC_Os07g26560	0.36	0.37	conserved hypothetical protein
LOC_Os03g28190	0.38	0.44	dirigent, putative, expressed
LOC_Os03g38960	0.39	0.32	DNA-directed RNA polymerase subunit, putative, expressed
LOC_Os03g30950	0.45	0.40	acyl-desaturase, chloroplast precursor, putative, expressed
LOC_Os03g30300	0.46	0.39	6-phosphogluconolactonase, putative, expressed
LOC_Os08g06100	0.52	0.35	O-methyltransferase, putative, expressed
LOC_Os03g28330	0.57	0.54	sucrose synthase, putative, expressed

*These genes located in Chr. 3 but not the segments harbors the different segments from Koshihikari (from RM15144 to RM411 positions from Chr3. 17,679,472 to 21,231,694) or N411 (from XJ19 to RM15575 positions from Chr3. 24953205 to 25099651) checked with MSU Rice Genome Annotation (Osa1) Release 7.

Supplemental Table S3 Genes up-regulated by *qgl3* (>1.5-fold)

MSU_Gene_Symbol*	*qgl3*/*qGL3* (*GS3*;fold)	*qgl3*/*qGL3* (*gs3*;fold)	Discription
LOC_Os02g56310	32.63	53.03	calcium-dependent protein kinase isoform AK1, putative, expressed
LOC_Os11g47610	12.37	5.03	glycosyl hydrolase, putative, expressed
LOC_Os08g04240	6.69	23.33	cysteine-rich repeat secretory protein 55 precursor, putative, expressed
LOC_Os07g34570	2.09	16.86	FAD dependent oxidoreductase domain containing protein, expressed
LOC_Os01g62130	2.53	6.61	ZOS1-14 - C2H2 zinc finger protein, expressed
LOC_Os02g54130	3.29	5.87	heat shock protein DnaJ, putative, expressed
LOC_Os02g51620	3.46	5.65	glycosyl hydrolase family 3 protein, putative, expressed
LOC_Os02g55770	4.2	3.85	expressed protein
LOC_Os04g28420	7.62	17.97	peptidyl-prolyl isomerase, putative, expressed
LOC_Os01g08860	1.79	9.47	hsp20/alpha crystallin family

			protein, putative, expressed
LOC_Os08g17500	2.4	2.73	cinnamoyl-CoA reductase, putative, expressed
LOC_Os02g55720	2.6	2.54	cyclin, N-terminal domain containing protein
LOC_Os02g52220	2.4	2.62	PPR repeat domain containing protein, putative, expressed
LOC_Os02g52190	1.92	2.72	helix-loop-helix DNA-binding protein, putative, expressed
LOC_Os02g52960	3.14	2.75	PHD-finger domain containing protein, putative, expressed
LOC_Os02g54700	2.13	2.55	RNA recognition motif containing protein, putative, expressed
LOC_Os02g11070	1.88	2.49	3-ketoacyl-CoA synthase, putative, expressed
LOC_Os02g56510	3.06	3.01	phosphate transporter 1, putative, expressed
LOC_Os02g52130	2.22	3.08	OsFBX61 - F-box domain containing protein, expressed
LOC_Os11g45400	3.07	1.97	glycerol-3-phosphate acyltransferase, putative, expressed
LOC_Os02g52360	1.92	4.1	peptidyl-prolyl cis-trans isomerase CYP40, putative, expressed
LOC_Os02g51710	2.09	2.9	wound/stress protein, putative, expressed
LOC_Os02g56480	1.96	1.87	PB1 domain containing protein,

			expressed
LOC_Os03g22590	2.78	2.03	nodulin MtN3 family protein, putative, expressed
LOC_Os02g51590	1.9	2.03	phosphoglycerate mutase, putative, expressed
LOC_Os09g36700	2.43	1.88	ribonuclease T2 family domain containing protein, expressed
LOC_Os02g55140	1.8	1.71	leucine aminopeptidase, chloroplast precursor, putative, expressed
LOC_Os02g55134	1.61	2.1	cytochrome c oxidase copper chaperone, putative, expressed
LOC_Os02g56170	1.7	2.43	inositol-1-monophosphatase, putative, expressed
LOC_Os02g54150	1.92	1.95	SAC domain containing protein, putative, expressed
LOC_Os01g08700	2.41	2.08	GIGANTEA, putative, expressed
LOC_Os05g35470	1.59	2.27	dienelactone hydrolase family protein, expressed
LOC_Os06g42860	1.95	2.91	triacylglycerol lipase precursor, putative, expressed

*These genes located in Chr. 3 but not the segments harbors the different segments from Koshihikari (from RM15144 to RM411 positions from Chr3. 17,679,472 to 21,231,694) or N411 (from XJ19 to RM15575 positions from Chr3. 24953205 to 25099651) checked with MSU Rice Genome Annotation (Osa1) Release 7.

Supplemental Table S4 Genes down-regulated by *qgl3* (<0.67-fold)

MSU_Gene_Symbol*	*qgl3*/*qGL3* (*GS3*;fold)	*qgl3*/*qGL3* (*gs3*;fold)	Discription

LOC_Os02g55540	0.24	0.27	F-box/LRR-repeat protein 14, putative, expressed
LOC_Os01g60340	0.30	0.36	NTMC2Type1.1 protein, putative
LOC_Os07g01840	0.56	0.49	expressed protein
LOC_Os07g44060	0.45	0.51	haloacid dehalogenase-like hydrolase family protein, putative, expressed
LOC_Os08g36540	0.54	0.41	aspartic proteinase nepenthesin-1 precursor, putative, expressed
LOC_Os02g51790	0.40	0.49	ribosomal protein L29, putative, expressed
LOC_Os06g45450	0.37	0.41	glucan endo-1,3-beta-glucosidase 12 precursor, putative
LOC_Os11g47944	0.57	0.60	thaumatin, putative
LOC_Os02g54530	0.37	0.61	SCP-like extracellular protein, expressed
LOC_Os07g35520	0.57	0.53	glucan endo-1,3-beta-glucosidase precursor, putative, expressed
LOC_Os12g39630	0.53	0.51	CAMK_CAMK_like.49 - CAMK includes calcium/calmodulin depedent protein kinases, expressed
LOC_Os04g49450	0.37	0.31	MYB family transcription factor, putative, expressed
LOC_Os01g48130	0.37	0.64	no apical meristem protein, putative, expressed
LOC_Os09g32770	0.48	0.67	auxin efflux carrier component, putative, expressed
LOC_Os03g62020	0.48	0.52	harpin-induced protein 1 domain containing protein, expressed
LOC_Os02g56000	0.41	0.66	26S protease regulatory subunit 6A, putative, expressed
LOC_Os07g01530	0.50	0.44	expressed protein

LOC_Os03g43410	0.40	0.51	OsIAA12 - Auxin-responsive Aux/IAA gene family member, expressed
LOC_Os02g49560	0.61	0.64	bZIP transcription factor domain containing protein, expressed
LOC_Os08g35760	0.57	0.52	Cupin domain containing protein, expressed
LOC_Os02g55260	0.46	0.54	DEAD-box ATP-dependent RNA helicase, putative, expressed
LOC_Os10g39740	0.41	0.52	glutathione S-transferase, putative, expressed
LOC_Os01g44090	0.61	0.66	X8 domain containing protein, expressed
LOC_Os06g43030	0.62	0.62	protein kinase domain containing protein, expressed
LOC_Os03g44540	0.37	0.60	nuclear transcription factor Y subunit, putative, expressed
LOC_Os02g50330	0.58	0.56	RNA-dependent RNA polymerase, putative, expressed
LOC_Os08g01780	0.55	0.62	OsIAA25 - Auxin-responsive Aux/IAA gene family member, expressed
LOC_Os12g24040	0.52	0.62	glycosyl hydrolase family 9 protein, expressed
LOC_Os10g40420	0.49	0.43	LTPL138 - Protease inhibitor/seed storage/LTP family protein precursor, expressed
LOC_Os05g02310	0.53	0.61	soluble inorganic pyrophosphatase, putative, expressed

*These genes located in Chr. 3 but not the segments harbors the different segments from Koshihikari (from RM15144 to RM411 positions from Chr3. 17,679,472 to 21,231,694) or N411 (from XJ19 to RM15575 positions from Chr3. 24953205 to 25099651) checked with MSU Rice Genome Annotation (Osa1) Release 7.

Supplemental Table S5 Genes up-regulated by both *qgl3* and *gs3* (> 1.5-fold).

MSU_Gene_Symbol*	*qgl3*/*qGL3* (fold)	*gs3*/*GS3* (fold)	Description
LOC_Os03g42370	5.63	9.89	B3 DNA binding domain containing protein
LOC_Os03g40400	2.68	3.56	interferon-induced, double-stranded RNA-activated protein kinase, putative
LOC_Os11g44880	2.42	2.24	kinesin-4, putative, expressed
LOC_Os01g59990	2.28	2.08	ribosomal protein L24, putative, expressed
LOC_Os01g60280	2.02	2.20	ATP binding protein, putative, expressed
LOC_Os11g47460	2.30	2.10	MYB family transcription factor, putative, expressed
LOC_Os03g43800	1.82	2.32	DIRP family protein, putative, expressed
LOC_Os02g52930	1.73	1.54	integral membrane protein DUF6 containing protein, expressed
LOC_Os03g40020	1.65	1.98	PPR repeat containing protein, expressed
LOC_Os03g64050	1.54	1.80	receptor protein kinase, putative, expressed
LOC_Os04g59000	1.51	1.59	protein kinase family protein, putative, expressed

*These genes located in Chr. 3 but not the segments harbors the different segments from Koshihikari (from RM15144 to RM411 positions from Chr3. 17,679,472 to 21,231,694) or N411 (from XJ19 to RM15575 positions from Chr3. 24953205 to 25099651) checked with MSU Rice Genome Annotation (Osa1) Release 7.

Supplemental Table S6 Genes down-regulated by both *qgl3* and *gs3* (<0.67-fold).

MSU_Gene_Symbol*	*qgl3*/*qGL3* (fold)	*gs3*/*GS3* (fold)	Descriptions
LOC_Os07g43670	0.03	0.02	ribonuclease T2 family domain containing protein, expressed
LOC_Os11g45990	0.04	0.03	von Willebrand factor type A domain containing protein, putative, expressed
LOC_Os05g07090	0.09	0.04	acyl-coenzyme A dehydrogenase, mitochondrial precursor, putative, expressed
LOC_Os11g47447	0.07	0.07	stripe rust resistance protein Yr10, putative, expressed
LOC_Os11g43980	0.15	0.09	peroxidase precursor, putative, expressed
LOC_Os11g45790	0.14	0.15	NB-ARC domain containing protein, expressed
LOC_Os03g43820	0.26	0.16	plant invertase/pectin methylesterase inhibitor domain containing protein, expressed
LOC_Os11g46210	0.12	0.17	NB-ARC domain containing protein, expressed
LOC_Os03g40830	0.41	0.17	OsSub30 - Putative Subtilisinhomologue, expressed
LOC_Os03g36530	0.53	0.19	FAD-binding and arabino-lactone oxidase domains containing protein, putative, expressed
LOC_Os11g44600	0.17	0.19	calmodulin binding protein, putative, expressed
LOC_Os11g45280	0.21	0.19	protein kinase family protein, putative, expressed
LOC_Os01g60790	0.26	0.21	40S ribosomal protein S26, putative, expressed

LOC_Os09g07290	0.39	0.22	GDSL-like lipase/acylhydrolase, putative, expressed
LOC_Os03g59330	0.22	0.23	polygalacturonase, putative, expressed
LOC_Os11g44560	0.22	0.24	protein kinase domain containing protein, expressed
LOC_Os03g63200	0.53	0.27	powdery mildew resistance protein PM3b, putative, expressed
LOC_Os03g40070	0.52	0.27	transposon protein, putative, unclassified, expressed
LOC_Os01g56530	0.30	0.28	DUF260 domain containing protein, putative, expressed
LOC_Os03g63970	0.54	0.29	gibberellin 20 oxidase 1, putative, expressed
LOC_Os01g60770	0.48	0.31	expansin precursor, putative, expressed
LOC_Os03g63870	0.37	0.33	expressed protein
LOC_Os04g05700	0.40	0.35	expressed protein
LOC_Os03g64150	0.63	0.35	MATE efflux family protein, putative, expressed
LOC_Os02g44108	0.55	0.39	expansin precursor, putative, expressed
LOC_Os04g05770	0.54	0.40	conserved hypothetical protein
LOC_Os09g37420	0.30	0.40	OsSAUR47 - Auxin-responsive SAUR gene family member, expressed
LOC_Os02g13290	0.46	0.41	phosphoethanolamine/phosphocholine phosphatase, putative, expressed
LOC_Os11g47140	0.35	0.41	OsWAK123 - OsWAK receptor-like protein kinase, expressed
LOC_Os11g48090	0.54	0.44	helicase conserved C-terminal domain containing protein, expressed
LOC_Os01g61780	0.49	0.44	vacuolar ATP synthase 98 kDa subunit, putative, expressed

LOC_Os01g64960	0.65	0.46	chlorophyll A-B binding protein, putative, expressed
LOC_Os04g59060	0.46	0.47	heat shock protein DnaJ, putative, expressed
LOC_Os07g30620	0.30	0.48	cytokinin-O-glucosyltransferase 2, putative, expressed
LOC_Os01g60170	0.50	0.49	DUF567 domain containing protein, putative, expressed
LOC_Os06g08250	0.66	0.50	zinc finger family protein, putative, expressed
LOC_Os01g14670	0.48	0.50	Cupin domain containing protein, expressed
LOC_Os10g17790	0.35	0.51	remorin C-terminal domain containing protein, putative, expressed
LOC_Os05g04340	0.52	0.51	CGMC_GSK.6 - CGMC includes CDA, MAPK, GSK3, and CLKC kinases, expressed
LOC_Os09g38920	0.48	0.51	thiol protease SEN102 precursor, putative, expressed
LOC_Os03g08500	0.56	0.52	AP2 domain containing protein, expressed
LOC_Os01g62370	0.44	0.53	ENTH domain containing protein, expressed
LOC_Os01g60440	0.51	0.53	HEAT repeat family protein, putative, expressed
LOC_Os12g12580	0.40	0.53	NADP-dependent oxidoreductase, putative, expressed
LOC_Os01g52230	0.58	0.53	phosphoethanolamine/phosphocholine phosphatase, putative, expressed
LOC_Os02g47020	0.43	0.53	phosphoribulokinase/Uridine kinase family protein, expressed
LOC_Os03g42230	0.37	0.53	B3 DNA binding domain containing protein, expressed
LOC_Os07g08160	0.51	0.55	early light-induced protein, chloroplast precursor, putative, expressed

LOC_Os02g20170	0.57	0.56	drought induced 19 protein, putative, expressed
LOC_Os06g51290	0.65	0.57	phytoene synthase, chloroplast precursor, putative, expressed
LOC_Os03g14590	0.60	0.58	calcium-binding EF hand family protein, putative, expressed
LOC_Os03g16090	0.54	0.59	LIM domain-containing protein, putative, expressed
LOC_Os11g44750	0.56	0.59	retrotransposon protein, putative, unclassified
LOC_Os08g34280	0.58	0.60	cinnamoyl-CoA reductase, putative, expressed
LOC_Os04g06850	0.56	0.60	expressed protein
LOC_Os01g62244	0.65	0.61	ubiquitin-conjugating enzyme, putative, expressed
LOC_Os03g43840	0.65	0.61	LSD1 zinc finger domain containing protein, expressed
LOC_Os05g51160	0.61	0.61	Myb transcription factor, putative, expressed
LOC_Os06g14670	0.53	0.62	ODORANT1, putative, expressed
LOC_Os01g47630	0.46	0.62	expressed protein
LOC_Os04g46130	0.45	0.64	plastocyanin-like domain containing protein, putative, expressed
LOC_Os04g59620	0.61	0.64	SNF2 family N-terminal domain containing protein, expressed
LOC_Os03g12270	0.60	0.66	dehydrogenase, putative, expressed
LOC_Os05g31280	0.52	0.66	GASR5-Gibberellin-regulated GASA/GAST/Snak in family protein precursor, expressed

*These genes located in Chr. 3 but not the segments harbors the different segments from Koshihikari (from RM15144 to RM411 positions from Chr3. 17,679,472 to

21,231,694) or N411 (from XJ19 to RM15575 positions from Chr3. 24953205 to 25099651) checked with MSU Rice Genome Annotation (Osa1) Release 7.

Supplemental Table S7 *qGL3* × *GS3* interactions resolved by two-way ANOVA for expression levels of commonly down-regulated genes.

MSU_Gene_Symbol*	Variation	SS	*df*	MS	*F*	*P* value
LOC_Os09g07290	*qGL3*	2498	1	2498	392.5	4.39E-08
	GS3	1060	1	1060	166.5	1.23E-06
	qGL3×*GS3*	3931	1	3931	617.6	7.35E-09
	Error	51	8	6		
LOC_Os11g44600	*qGL3*	709	1	709	582.0	9.29E-09
	GS3	892	1	892	732.4	3.74E-09
	qGL3×*GS3*	593	1	593	486.5	1.89E-08
	Error	10	8	1		
LOC_Os10g17790	*qGL3*	2367	1	2367	474.4	2.08E-08
	GS3	3707	1	3707	742.9	3.54E-09
	qGL3×*GS3*	780	1	780	156.3	1.57E-06
	Error	40	8	5		
LOC_Os11g48090	*qGL3*	47377	1	47377	583.9	9.18E-09
	GS3	38104	1	38104	469.6	2.17E-08
	qGL3×*GS3*	49737	1	49737	612.9	7.57E-09
	Error	649	8	81		
LOC_Os11g45280	*qGL3*	259004	1	259004	11132.4	7.27E-14
	GS3	256614	1	256614	11029.6	7.55E-14
	qGL3×*GS3*	203956	1	203956	8766.3	1.89E-13
	Error	186	8	23		
LOC_Os01g61780	*qGL3*	141540	1	141540	2084.6	5.85E-11
	GS3	111107	1	111107	1636.4	1.53E-10
	qGL3×*GS3*	102640	1	102640	1511.7	2.1E-10

	Error	543	8	68		
LOC_Os01g60440	*qGL3*	287164	1	287164	375.0	5.25E-08
	GS3	160329	1	160329	209.4	5.09E-07
	qGL3×*GS3*	271168	1	271168	354.1	6.57E-08
	Error	6126	8	766		
LOC_Os01g56530	*qGL3*	15085	1	15085	378.7	5.05E-08
	GS3	17918	1	17918	449.8	2.57E-08
	qGL3×*GS3*	10188	1	10188	255.7	2.34E-07
	Error	319	8	40		
LOC_Os02g13290	*qGL3*	350	1	350	61.0	5.18E-05
	GS3	209	1	209	36.4	0.000313
	qGL3×*GS3*	313	1	313	54.5	7.75E-05
	Error	46	8	6		
LOC_Os03g16090	*qGL3*	15744	1	15744	766.1	3.13E-09
	GS3	26059	1	26059	1267.9	4.24E-10
	qGL3×*GS3*	8882	1	8882	432.2	3.01E-08
	Error	164	8	21		
LOC_Os03g14590	*qGL3*	238047	1	238047	145.0	2.09E-06
	GS3	273426	1	273426	166.6	1.23E-06
	qGL3×*GS3*	281746	1	281746	171.6	1.1E-06
	Error	13132	8	1641		
LOC_Os03g64150	*qGL3*	5495	1	5495	412.7	3.6E-08
	GS3	278	1	278	20.8	0.001836
	qGL3×*GS3*	4195	1	4195	315.1	1.04E-07
	Error	107	8	13		
LOC_Os04g59620	*qGL3*	217555	1	217555	1621.3	1.59E-10
	GS3	173123	1	173123	1290.2	3.95E-10
	qGL3×*GS3*	20434	1	20434	152.3	1.73E-06
	Error	1073	8	134		

LOC_Os06g14670	*qGL3*	12795	1	12795	352.5	6.69E-08
	GS3	24450	1	24450	673.6	5.21E-09
	qGL3×*GS3*	7634	1	7634	210.3	5E-07
	Error	290	8	36		
LOC_Os07g43670	*qGL3*	263606	1	263606	18743.8	9.06E-15
	GS3	256860	1	256860	18264.1	1E-14
	qGL3×*GS3*	253967	1	253967	18058.4	1.05E-14
	Error	113	8	14		
LOC_Os11g45990	*qGL3*	2335	1	2335	297.0	1.31E-07
	GS3	2611	1	2611	332.1	8.45E-08
	qGL3×*GS3*	1928	1	1928	245.2	2.76E-07
	Error	63	8	8		
LOC_Os11g47447	*qGL3*	31272	1	31272	2840.5	1.7E-11
	GS3	29874	1	29874	2713.5	2.04E-11
	qGL3×*GS3*	28197	1	28197	2561.2	2.57E-11
	Error	88	8	11		
LOC_Os11g44560	*qGL3*	490	1	490	111.9	5.58E-06
	GS3	649	1	649	148.1	1.93E-06
	qGL3×*GS3*	395	1	395	90.1	1.25E-05
	Error	35	8	4		
LOC_Os11g43980	*qGL3*	24767	1	24767	2157.1	5.1E-11
	GS3	21729	1	21729	1892.5	8.6E-11
	qGL3×*GS3*	41505	1	41505	3614.9	6.51E-12
	Error	92	8	11		
LOC_Os11g45790	*qGL3*	5619	1	5619	2017.1	6.67E-11
	GS3	7090	1	7090	2545.1	2.64E-11
	qGL3×*GS3*	5766	1	5766	2069.7	6.02E-11
	Error	22	8	3		
LOC_Os01g62370	*qGL3*	452	1	452	211.7	4.87E-07

	GS3	834	1	834	390.8	4.47E-08
	qGL3×*GS3*	183	1	183	85.6	1.51E-05
	Error	17	8	2		
LOC_Os01g60790	*qGL3*	405737	1	405737	767.3	3.11E-09
	GS3	274277	1	274277	518.7	1.46E-08
	qGL3×*GS3*	506864	1	506864	958.5	1.29E-09
	Error	4230	8	529		
LOC_Os01g60770	*qGL3*	780670	1	780670	469.5	2.17E-08
	GS3	278475	1	278475	167.5	1.2E-06
	qGL3×*GS3*	1407210	1	1407210	846.2	2.11E-09
	Error	13303	8	1663		
LOC_Os01g62244	*qGL3*	201917	1	201917	242.6	2.88E-07
	GS3	381561	1	381561	458.3	2.38E-08
	qGL3×*GS3*	203840	1	203840	244.9	2.77E-07
	Error	6660	8	832		
LOC_Os02g44108	*qGL3*	124304	1	124304	193.9	6.85E-07
	GS3	78557	1	78557	122.5	3.96E-06
	qGL3×*GS3*	686901	1	686901	1071.3	8.28E-10
	Error	5129	8	641		
LOC_Os03g63970	*qGL3*	360138	1	360138	1377.4	3.05E-10
	GS3	83769	1	83769	320.4	9.73E-08
	qGL3×*GS3*	273966	1	273966	1047.8	9.04E-10
	Error	2092	8	261		
